# Supplementary material for: Machine Learning Accelerates Crystallization for Structure Determination
Source: Angew Chem Int Ed Engl. 2026 May 4;65(25):e1218503. doi: 10.1002/anie.1218503 (PMC13266958; doi:10.1002/anie.1218503)

checkCIF/PLATON report

Structure factors have been supplied for datablock(s) lcz082

THIS REPORT IS FOR GUIDANCE ONLY. IF USED AS PART OF A REVIEW PROCEDURE FOR PUBLICATION, IT SHOULD NOT REPLACE THE EXPERTISE OF AN EXPERIENCED CRYSTALLOGRAPHIC REFEREE.

No syntax errors found. CIF dictionary Interpreting this report

Datablock: lcz082

Bond precision:	C-C = 0.0245 A		Wavelength=1.54184
Cell:	a=27.4648(11)	b=12.8992(5)	c=28.1210(15)
	alpha=90	beta=97.239(4)	gamma=90
Temperature:	100 K		

	Calculated	Reported
Volume	9883.1(8)	9883.1(8)
Space group	C 2/c	C 1 2/c 1
Hall group	-C 2yc	-C 2yc
Moiety formula	3(C15 H3 Ag3 F18 N6), 2(C12 H9 N) [+ solvent]	C15 H3 Ag3 F18 N6, 0.667(C12 H9 N)
Sum formula	C69 H27 Ag9 F54 N20 [+ solvent]	C23 H9 Ag3 F18 N6.67
Mr	3132.94	1044.32
Dx,g cm-3	2.106	2.106
Z	4	12
Mu (mm-1)	15.395	15.395
F000	5960.0	5960.0
F000'	5989.45	
h,k,lmax	34,16,35	33,16,33
Nref	10602	9929
Tmin,Tmax	0.115,0.214	0.453,1.000
Tmin'	0.018	

```
Correction method= # Reported T Limits: Tmin=0.453 Tmax=1.000
AbsCorr = MULTI-SCAN

Data completeness= 0.937            Theta(max)= 78.134

R(reflections)= 0.1062( 8437)       wR2(reflections)= 0.2734( 9929)

S = 1.026                 Npar= 677
```

The following ALERTS were generated. Each ALERT has the format
test-name_ALERT_alert-type_alert-level.
Click on the hyperlinks for more details of the test.

Alert level B

```
PLAT342_ALERT_3_B Low Bond Precision on  C-C Bonds ..............    0.02453 Ang.
```

Author Response: Disordered structure.

```
PLAT972_ALERT_2_B Check Calcd Resid. Dens.  0.81Ang From Ag5            -2.94 eA-3
```

Author Response: This Alert is due to presence of residual density in the presence of heavy metal atom (Ag).

```
PLAT972_ALERT_2_B Check Calcd Resid. Dens.  0.70Ang From Ag4            -2.56 eA-3
```

Author Response: This Alert is due to presence of residual density in the presence of heavy metal atom (Ag).

Alert level C

```
PLAT082_ALERT_2_C High R1 Value ................................       0.11 Report
PLAT084_ALERT_3_C High wR2 Value (i.e. > 0.25) ..................       0.27 Report
PLAT230_ALERT_2_C Hirshfeld Test Diff for    F00X     --C01S     .        6.2 s.u.
PLAT230_ALERT_2_C Hirshfeld Test Diff for    F026     --C025     .        5.5 s.u.
PLAT234_ALERT_4_C Large Hirshfeld Difference F00I     --C01M     .       0.20 Ang.
PLAT234_ALERT_4_C Large Hirshfeld Difference F00Y     --C01X     .       0.23 Ang.
PLAT234_ALERT_4_C Large Hirshfeld Difference N4       --N5       .       0.17 Ang.
PLAT234_ALERT_4_C Large Hirshfeld Difference C01E     --C019     .       0.17 Ang.
PLAT234_ALERT_4_C Large Hirshfeld Difference F01R     --C02A     .       0.19 Ang.
PLAT234_ALERT_4_C Large Hirshfeld Difference F014     --C025     .       0.20 Ang.
PLAT234_ALERT_4_C Large Hirshfeld Difference C01C     --C025     .       0.17 Ang.
PLAT234_ALERT_4_C Large Hirshfeld Difference C02A     --C022     .       0.21 Ang.
PLAT234_ALERT_4_C Large Hirshfeld Difference C020     --C022     .       0.22 Ang.
PLAT420_ALERT_2_C D-H Bond Without Acceptor  N10      --H10      .     Please Check
PLAT906_ALERT_3_C Large K Value in the Analysis of Variance ......      6.153 Check
PLAT906_ALERT_3_C Large K Value in the Analysis of Variance ......      2.273 Check
PLAT911_ALERT_3_C Missing FCF Refl Between Thmin & STh/L=    0.600        144 Report
```

```
                 2   2   0,    4   2   0,    5   3   0,   16   0   0,   -4   4   1,   -1   7   1,
                 1   1   1, -16   0   2,   -5   3   2,   -1   7   2,    2   0   2,    2   6   2,
                 2   8   2,    3   9   2,    1   1   3,    5   1   3,   15   1   3,    4   0   4,
                 4   2   4,    6   2   4,   12  14   4,    3   1   5,   10  14   5,   -9   1   6,
                 2   2   6,    6   0   6, -12   4   7,    2   6   7, -32   0   8, -11   3   8,
                   (  114 More Missing: see the .ckf listing file)
PLAT971_ALERT_2_C Check Calcd Resid. Dens.  0.98Ang From N2            2.34 eA-3
PLAT971_ALERT_2_C Check Calcd Resid. Dens.  0.89Ang From F01R          2.34 eA-3
PLAT971_ALERT_2_C Check Calcd Resid. Dens.  1.00Ang From Ag1           2.33 eA-3
PLAT971_ALERT_2_C Check Calcd Resid. Dens.  0.98Ang From Ag4           2.13 eA-3
PLAT971_ALERT_2_C Check Calcd Resid. Dens.  0.84Ang From Ag5           2.13 eA-3
PLAT971_ALERT_2_C Check Calcd Resid. Dens.  0.91Ang From Ag5           2.09 eA-3
PLAT971_ALERT_2_C Check Calcd Resid. Dens.  0.94Ang From Ag4           2.07 eA-3
PLAT971_ALERT_2_C Check Calcd Resid. Dens.  1.09Ang From Ag3           2.03 eA-3
PLAT971_ALERT_2_C Check Calcd Resid. Dens.  1.09Ang From Ag4           1.96 eA-3
PLAT971_ALERT_2_C Check Calcd Resid. Dens.  0.80Ang From F014          1.90 eA-3
PLAT971_ALERT_2_C Check Calcd Resid. Dens.  0.73Ang From F01R          1.89 eA-3
PLAT971_ALERT_2_C Check Calcd Resid. Dens.  0.98Ang From Ag2           1.87 eA-3
PLAT971_ALERT_2_C Check Calcd Resid. Dens.  0.94Ang From Ag3           1.83 eA-3
PLAT971_ALERT_2_C Check Calcd Resid. Dens.  0.91Ang From Ag1           1.79 eA-3
PLAT971_ALERT_2_C Check Calcd Resid. Dens.  0.90Ang From Ag2           1.77 eA-3
PLAT971_ALERT_2_C Check Calcd Resid. Dens.  0.82Ang From F00W          1.76 eA-3
PLAT971_ALERT_2_C Check Calcd Resid. Dens.  0.67Ang From F012          1.74 eA-3
PLAT971_ALERT_2_C Check Calcd Resid. Dens.  0.93Ang From Ag4           1.73 eA-3
PLAT971_ALERT_2_C Check Calcd Resid. Dens.  1.18Ang From Ag4           1.71 eA-3
PLAT971_ALERT_2_C Check Calcd Resid. Dens.  0.94Ang From Ag2           1.66 eA-3
PLAT971_ALERT_2_C Check Calcd Resid. Dens.  1.01Ang From Ag4           1.63 eA-3
PLAT971_ALERT_2_C Check Calcd Resid. Dens.  0.95Ang From Ag2           1.63 eA-3
PLAT971_ALERT_2_C Check Calcd Resid. Dens.  0.81Ang From F00W          1.61 eA-3
PLAT971_ALERT_2_C Check Calcd Resid. Dens.  1.22Ang From Ag2           1.58 eA-3
PLAT971_ALERT_2_C Check Calcd Resid. Dens.  0.95Ang From Ag1           1.58 eA-3
PLAT972_ALERT_2_C Check Calcd Resid. Dens.  0.91Ang From Ag2          -2.37 eA-3
```

Author Response: This Alert is due to presence of residual density in the presence of heavy metal atom (Ag).

```
PLAT972_ALERT_2_C Check Calcd Resid. Dens.  0.86Ang From Ag1          -2.37 eA-3
```

Author Response: This Alert is due to presence of residual density in the presence of heavy metal atom (Ag).

```
PLAT972_ALERT_2_C Check Calcd Resid. Dens.  0.79Ang From Ag3          -2.32 eA-3
```

Author Response: This Alert is due to presence of residual density in the presence of heavy metal atom (Ag).

```
PLAT972_ALERT_2_C Check Calcd Resid. Dens.  0.80Ang From Ag4          -2.22 eA-3
```

Author Response: This Alert is due to presence of residual density in the presence of heavy metal atom (Ag).

PLAT972_ALERT_2_C Check Calcd Resid. Dens. 0.82Ang From Ag2   -1.94 eA-3

Author Response: This Alert is due to presence of residual density in the presence of heavy metal atom (Ag).

PLAT972_ALERT_2_C Check Calcd Resid. Dens. 0.81Ang From Ag3   -1.93 eA-3

Author Response: This Alert is due to presence of residual density in the presence of heavy metal atom (Ag).

PLAT972_ALERT_2_C Check Calcd Resid. Dens. 0.67Ang From Ag4   -1.87 eA-3

Author Response: This Alert is due to presence of residual density in the presence of heavy metal atom (Ag).

PLAT972_ALERT_2_C Check Calcd Resid. Dens. 0.78Ang From Ag1   -1.76 eA-3

Author Response: This Alert is due to presence of residual density in the presence of heavy metal atom (Ag).

PLAT972_ALERT_2_C Check Calcd Resid. Dens. 0.86Ang From Ag2   -1.67 eA-3

Author Response: This Alert is due to presence of residual density in the presence of heavy metal atom (Ag).

PLAT972_ALERT_2_C Check Calcd Resid. Dens. 0.84Ang From Ag1   -1.63 eA-3

Author Response: This Alert is due to presence of residual density in the presence of heavy metal atom (Ag).

PLAT972_ALERT_2_C Check Calcd Resid. Dens. 0.63Ang From Ag4   -1.60 eA-3

Author Response: This Alert is due to presence of residual density in the presence of heavy metal atom (Ag).

PLAT972_ALERT_2_C Check Calcd Resid. Dens. 0.88Ang From Ag3   -1.54 eA-3

Author Response: This Alert is due to presence of residual density in the presence of heavy metal atom (Ag).

PLAT977_ALERT_2_C Check Negative Difference Density on H020   .   -0.43 eA-3

Alert level G

```
PLAT003_ALERT_2_G Number of Uiso or U(i,j)  Restrained non-H-Atoms         26 Report
PLAT007_ALERT_5_G Number of Unrefined Donor-H Atoms .............          1 Report
              H10
PLAT041_ALERT_1_G Calc. and Reported SumFormula    Strings  Differ     Please Check
              Calc: C69 H27 Ag9 F54 N20
              Rep.: C23 H9 Ag3 F18 N6.67
PLAT042_ALERT_1_G Calc. and Reported MoietyFormula Strings  Differ     Please Check
              Calc: 3(C15 H3 Ag3 F18 N6), 2(C12 H9 N)
              Rep.: C15 H3 Ag3 F18 N6, 0.667(C12 H9 N)
PLAT045_ALERT_1_G Calculated and Reported Z Differ by a Factor ...      0.333 Check
PLAT072_ALERT_2_G SHELXL First  Parameter in WGHT  Unusually Large       0.12 Report
PLAT083_ALERT_2_G SHELXL Second Parameter in WGHT  Unusually Large     568.83 Why ?
PLAT177_ALERT_4_G The CIF-Embedded .res File Contains DELU Records          4 Report
PLAT186_ALERT_4_G The CIF-Embedded .res File Contains ISOR Records          3 Report
PLAT192_ALERT_3_G A Non-default DELU Restraint Value for SecondPar     0.0200 Report
PLAT192_ALERT_3_G A Non-default DELU Restraint Value for SecondPar     0.0200 Report
PLAT192_ALERT_3_G A Non-default DELU Restraint Value for SecondPar     0.0200 Report
PLAT192_ALERT_3_G A Non-default DELU Restraint Value for SecondPar     0.0200 Report
PLAT242_ALERT_2_G Low    'MainMol' Ueq as Compared to Neighbors of       C01B Check
PLAT242_ALERT_2_G Low    'MainMol' Ueq as Compared to Neighbors of       C01M Check
PLAT242_ALERT_2_G Low    'MainMol' Ueq as Compared to Neighbors of       C01S Check
PLAT242_ALERT_2_G Low    'MainMol' Ueq as Compared to Neighbors of       C01U Check
PLAT242_ALERT_2_G Low    'MainMol' Ueq as Compared to Neighbors of       C01X Check
PLAT242_ALERT_2_G Low    'MainMol' Ueq as Compared to Neighbors of       C01Y Check
PLAT242_ALERT_2_G Low    'MainMol' Ueq as Compared to Neighbors of       C01K Check
PLAT242_ALERT_2_G Low    'MainMol' Ueq as Compared to Neighbors of       C025 Check
PLAT434_ALERT_2_G Short Inter HL..HL Contact F012     ..F00C     .       2.78 Ang.
                                  x,1-y,1/2+z  =      6_566 Check
PLAT434_ALERT_2_G Short Inter HL..HL Contact F014     ..F015     .       2.81 Ang.
                                    x,-1+y,z  =      1_545 Check
PLAT605_ALERT_4_G Largest Solvent Accessible VOID in the Structure        253 A**3
PLAT720_ALERT_4_G Number of Unusual/Non-Standard Labels ..........         75 Note
              F00B    F00D    F00F    F00I    F00J    F00L    F00M    F00N
              F00R    F00S    F00U    F00X    F00Y    C00Z    C01A    C01B
              C01E    H01E    C01F    H01F    C01I    C01L    H01L    C01M
              C01S    C01T    C01U    C01X    C01Y    F008    F009    F011
              F012    C017    F018    C019    F00C    F00E    F00G    F00W
              F01R    C01C    C01G    C01K    C02A    F006    F014    F015
              C016    H016    C020    H020    C022    C025    F026    C01H
              H01H    C01J    H01J    C01N    H01N    C01Q    C01P    C01O
              C01V    C02B    H02B    C010    H010    C023    H023    C027
              H027    C029    H029
PLAT860_ALERT_3_G Number of Least-Squares Restraints ............         148 Note
PLAT868_ALERT_4_G ALERTS Due to the Use of _smtbx_masks Suppressed          ! Info
PLAT883_ALERT_1_G Absent Datum for _atom_sites_solution_primary ..     Please Do !
PLAT910_ALERT_3_G Missing FCF Reflection(s) Below Theta(Min)[Deg]=       4.03 Note
                1  1  0,   2  0  0,   0  0  2,
PLAT912_ALERT_4_G Missing # of FCF Reflections Above STh/L=  0.600        447 Note
PLAT933_ALERT_2_G Number of HKL-OMIT Records in Embedded .res File         19 Note
              -12  4 13,  -9  1  6,  -6  2 15,  -4  4  1,   0  0  2,   1  1  3,
                2  0  0,   2  0  2,   2  2  0,   2  6  7,   3  1  5,   3  9  2,
                4  0  4,   4  2  4,   5  1  3,   6  0  6,   6  2  4,  12  0  8,
               17  7 22,
PLAT941_ALERT_3_G Average HKL Measurement Multiplicity ..........         2.2 Low
PLAT952_ALERT_5_G Calculated (ThMax) and CIF-Reported Lmax Differ.          2 Units
PLAT958_ALERT_1_G Calculated (ThMax) and Actual (FCF) Lmax Differ.          2 Units
PLAT969_ALERT_5_G The 'Henn et al.' R-Factor-gap value ..........      4.998 Note
```

```
              Predicted wR2: Based on SigI**2  5.47 or SHELX Weight 26.66
PLAT978_ALERT_2_G Number C-C Bonds with Positive Residual Density.         0 Info
```

```
   0 ALERT level A = Most likely a serious problem - resolve or explain
   3 ALERT level B = A potentially serious problem, consider carefully
  55 ALERT level C = Check. Ensure it is not caused by an omission or oversight
  36 ALERT level G = General information/check it is not something unexpected

   5 ALERT type 1 CIF construction/syntax error, inconsistent or missing data
  59 ALERT type 2 Indicator that the structure model may be wrong or deficient
  12 ALERT type 3 Indicator that the structure quality may be low
  15 ALERT type 4 Improvement, methodology, query or suggestion
   3 ALERT type 5 Informative message, check
```

Validation response form

Please find below a validation response form (VRF) that can be filled in and pasted into your CIF.

```
# start Validation Reply Form
_vrf_PLAT082_lcz082
;
PROBLEM: High R1 Value .................................       0.11 Report
RESPONSE: ...
;
_vrf_PLAT084_lcz082
;
PROBLEM: High wR2 Value (i.e. > 0.25) ..................       0.27 Report
RESPONSE: ...
;
_vrf_PLAT230_lcz082
;
PROBLEM: Hirshfeld Test Diff for    F00X     --C01S     .        6.2 s.u.
RESPONSE: ...
;
_vrf_PLAT234_lcz082
;
PROBLEM: Large Hirshfeld Difference F00I     --C01M     .       0.20 Ang.
RESPONSE: ...
;
_vrf_PLAT420_lcz082
;
PROBLEM: D-H Bond Without Acceptor  N10      --H10      .     Please Check
RESPONSE: ...
;
_vrf_PLAT906_lcz082
;
PROBLEM: Large K Value in the Analysis of Variance ......      6.153 Check
RESPONSE: ...
;
_vrf_PLAT911_lcz082
;
PROBLEM: Missing FCF Refl Between Thmin & STh/L=    0.600        144 Report
RESPONSE: ...
;
_vrf_PLAT971_lcz082
```

```
;
PROBLEM: Check Calcd Resid. Dens.  0.98Ang From N2              2.34 eA-3
RESPONSE: ...
;
_vrf_PLAT977_lcz082
;
PROBLEM: Check Negative Difference Density on H020      .      -0.43 eA-3
RESPONSE: ...
;
# end Validation Reply Form
```

It is advisable to attempt to resolve as many as possible of the alerts in all categories. Often the minor alerts point to easily fixed oversights, errors and omissions in your CIF or refinement strategy, so attention to these fine details can be worthwhile. It is up to the individual to critically assess their own results and, if necessary, seek expert advice.

PLATON version of 17/09/2025; check.def file version of 17/09/2025

duplicate check

No duplication found

Datablock lcz082 - ellipsoid plot

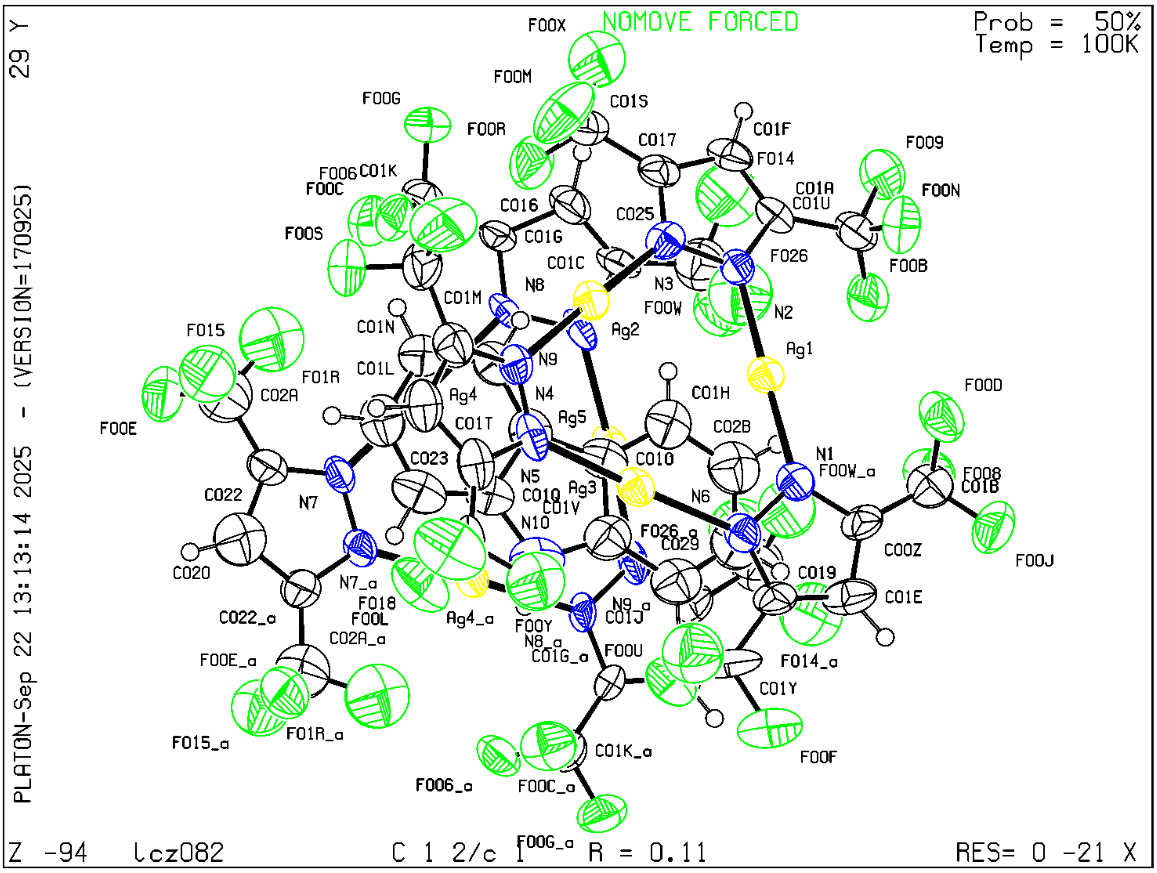

Supplement: Supplementary file 2 — Supporting File 2: anie72462‐sup‐0002‐DataFile.zip. [file ANIE-65-e1218503-s002.zip › supplementary_materials_crystallographic_data submit.zip!/cif_and_checkcif(1-114)/Ag3Pz3_82/Ag3Pz3_82_checkcif.pdf]
